# The Importance of Evaluating Sudomotor Function in the Diagnosis of Cardiac Autonomic Neuropathy

**DOI:** 10.7759/cureus.57226

**Published:** 2024-03-29

**Authors:** Andra E Nica, Emilia Rusu, Carmen G Dobjanschi, Florin Rusu, Oana A Parliteanu, Claudia Sivu, Gabriela Radulian

**Affiliations:** 1 Diabetes and Endocrinology, “Carol Davila” University of Medicine and Pharmacy, Bucharest, ROU; 2 Urology, “Doctor Carol Davila” Central Military University Emergency Hospital, Bucharest, ROU; 3 Diabetes and Endocrinology, Marius Nasta Institute of Pneumology, Bucharest, ROU; 4 Diabetes, “Carol Davila” University of Medicine and Pharmacy, Bucharest, ROU

**Keywords:** sudoscan, t2dm, ewing tests, sudomotor function, can

## Abstract

Introduction: Cardiac autonomic neuropathy (CAN) is a disorder affecting the autonomic nerves that regulate the cardiovascular system, leading to irregular heart rate and blood pressure control. It is commonly associated with diabetes mellitus but can also result from other conditions such as autoimmune disorders, chronic kidney disease, alcohol abuse, and certain medications. Screening for CAN is essential, particularly in individuals with poor glycemic control, cardiovascular risk factors, or complications. Early identification of CAN is vital for timely intervention to prevent or manage cardiovascular complications effectively. Regular screening helps detect CAN before symptoms emerge, enabling early intervention to slow or halt its progression. This study examined the relationship between sudomotor function and cardiovascular reflex tests.

Material and methods: This was a cross-sectional study conducted between June 2019 and June 2020. The study included 271 subjects aged 18 years and above who provided informed consent, were diagnosed with type 2 diabetes mellitus (T2DM), and were overweight or obese. Exclusion criteria encompassed patients with other types of diabetes, pregnant women, those with recent neoplasm diagnoses, stroke sequelae, history of myocardial infarction, or pelvic limb amputations. The assessment of cardiac autonomic neuropathy involved conducting an electrocardiogram and evaluating the QTc interval in the morning before taking medication. Additionally, cardiovascular reflex tests (CART) were conducted, including assessments of heart rate variability during deep breathing, the Valsalva maneuver, and changes in orthostatic position. Simultaneously, the diagnosis of CAN was assessed by performing a sweat test using a Sudoscan assessment (Impeto Medical, Moulineaux, France).

Results: More than half of the participants (52%, n=143) were female. Significant differences in statistical measures were noted between females and males regarding age, systolic blood pressure, fasting blood glucose, A1c level, total cholesterol, triglycerides, gamma-glutamyl transferase, and bilirubin levels. Within the CAN-diagnosed group (CAN+), 40.92% were classified as mild cases (n=90), 47.27% as moderate cases (n=104), and 11.81% as severe cases (n=26). Among the CAN+ group, 54% (n=119) were women. Electrochemical skin conductance was lower in the CAN+ group than the CAN- group in hands (67.34±15.51 μS versus 72.38±12.12 μS, p=0.008) and feet (73.37±13.38 μS versus 82.84 ±10.29 μS, p<0.001). The Sudoscan-CAN score significantly correlated with Ewing scores (r= 0.522, p<0.001). In multiple linear regression analysis, the Sudoscan-CAN score remained significantly associated with age, high BMI, long-standing diabetes, and Ewing score.

Conclusions:* *Sudoscan demonstrates potential in identifying patients with an increased risk of CAN. Its integration into clinical practice can improve patient outcomes through early detection, risk stratification, and personalized treatment approaches. Its non-invasive, portable, and user-friendly features render it suitable for utilization in outreach programs or resource-constrained settings as part of screening efforts designed to pinpoint high-risk individuals for additional assessment.

## Introduction

Cardiac autonomic neuropathy (CAN) is a disorder characterized by autonomic nerve dysfunction that governs the cardiovascular system, including heart rate, blood pressure, and vascular tone regulation. Damage to these nerves results in heart rate and blood pressure control irregularities. While often linked with diabetes mellitus, particularly in cases of prolonged inadequate glycemic control, CAN may also stem from other conditions like autoimmune disorders, chronic kidney disease, alcohol abuse, and specific medications. Despite the abundance of data linking type 2 diabetes mellitus (T2DM) with autonomic neuropathy, there is a growing body of evidence suggesting that CAN is prevalent among individuals with impaired glucose tolerance [1]. Screening for CAN is crucial, especially in individuals with a history of poor glycemic control, the presence of significant cardiovascular risk factors, or macro- or micro-angiopathic complications. Cardiovascular autonomic reflex tests (CARTs), a non-invasive and easily performed method, stand as the gold standard for diagnosing CAN [2]. Clinical assessment methods for CAN typically involve evaluating symptoms and signs. Common clinical presentations of CAN include orthostatic symptoms, tachycardia, exercise intolerance, orthostatic hypotension, QT prolongation, and abnormalities in ambulatory blood pressure monitoring, such as non-dipping or reverse dipping patterns [3]. Unfortunately, these clinical indicators have limited effectiveness in diagnosing CAN.

The prevalence of CAN in T2DM ranges from 20% to 73%. It represents the most perilous manifestation of diabetic autonomic neuropathy, carrying a significant independent risk for a grim prognosis. Patients face mortality rates between 27% and 56% over a 5-10-year follow-up period and an elevated susceptibility to sudden cardiac death [4]. Established risk factors for CAN include poor glycemic control in type 1 diabetes, while in T2DM, a combination of hypertension, dyslipidemia, obesity, and glycemic control contributes to its development. Managing glucose levels and blood pressure is crucial in preventing the progression of CAN [5].

CAN serves as a prognostic indicator for mortality and cardiovascular morbidity, and it may also exacerbate the progression of diabetic nephropathy. Diagnostic criteria and staging for CAN include (a) identification of one abnormal cardiovagal test result suggesting possible or early CAN, (b) confirmation of CAN requires at least two abnormal cardiovagal test results, and (c) the presence of orthostatic hypotension alongside abnormal heart rate test results signifies severe or advanced CAN [6]. The meta-analysis of 15 longitudinal studies published up to 2001 revealed that a confirmed diagnosis of CAN is linked to a relative mortality risk of 3.65 [7]. More recent studies, including large samples of patients, such as EURODIAB for type 1 diabetes and Action to Control Cardiovascular Risk in Diabetes (ACCORD) for T2DM, have documented through cardiovascular reflex testing that CAN is an independent predictor of cardiovascular mortality [8,9]. The mechanisms underlying the increased risk of mortality and morbidity associated with CAN remain largely unknown. Several cardiovascular abnormalities have been found in association with CAN. These may represent (a) a form of morbidity in itself, such as silent myocardial ischemia, (b) a recognized risk factor for mortality or morbidity (resting tachycardia, orthostatic hypotension, QT interval prolongation (QTc), baroreflex sensitivity impairment (BRS), non-dipping, reduced heart rate variability), and (c) an imbalance in sympathovagal activity, sympathetic denervation, decreased sympathetic-mediated vasodilation of the coronary arteries, as well as new mechanisms such as increased arterial stiffness and inflammation [10].

Resting tachycardia is the least specific sign of CAN and may also reflect vagal insufficiency and sympathetic hyperactivity present in cardiac diseases, obesity, or anemia. Tachycardia has prognostic value in both the general and diabetic populations. In the Action in Diabetes and Vascular Disease: Preterax and Diamicron MR Controlled Evaluation (ADVANCE) study involving 11,140 patients with T2DM, a higher resting heart rate was associated with an increased risk of all-cause mortality [11].

Orthostatic hypotension (OH) is defined as a sustained decrease in systolic blood pressure of 20 mm Hg or diastolic blood pressure of 10 mm Hg in normotensive subjects and a decrease in systolic blood pressure of 30 mm Hg in hypertensive subjects upon standing [12]. A meta-analysis of 13 observational studies, including 121,913 subjects with and without diabetes and hypertension, with a median follow-up of six years, showed that the prevalence of orthostatic hypotension was associated with an increased risk of death from ischemic coronary disease, heart failure, and stroke, with a pooled relative risk estimate for total mortality of 1.78 in patients younger than 65 years and 1.26 in the older subgroup. Similarly, in patients with diabetes mellitus, OH increases the risk of mortality associated with parasympathetic neuropathy by 30% to 100% [13].

Using ambulatory blood pressure monitoring with a Holter device, we can identify the non-dipping or reverse-dipping blood pressure profiles. A decrease in blood pressure from day to night of <10% identifies non-dipping and, respectively, reverse dipping [14]. Nondipping is more common in diabetic patients (39-62%) than in the general population, while reverse dipping is present in 9-30%.

In a study conducted on a population from China, He et al. concluded that the electrochemical skin conductance measurement is reliable and feasible for screening for CAN, among Chinese diabetic patients as a noninvasive, quantitative, and fast method, especially in routine clinical practice and large-scale epidemiological surveys before further diagnosis with cardiovascular reflex tests [15].

Although commonly linked with diabetes mellitus, CAN can also develop due to other medical conditions. CARTs are considered the standard diagnostic method. This complication is frequently observed in diabetes and is associated with notable mortality rates and an increased risk of sudden cardiac death. Effective management of blood sugar levels and blood pressure is critical in halting the progression of this disease.

Moreover, CAN serves as a valuable prognostic indicator for mortality and cardiovascular complications. Common clinical manifestations include resting tachycardia and orthostatic hypotension. Ambulatory blood pressure monitoring helps detect abnormal blood pressure fluctuations. Additionally, electrochemical skin conductance measurement shows promise as a reliable screening tool for CAN, particularly in routine clinical practice and large-scale surveys.

This study aimed to evaluate the correlation between sudomotor function and cardiovascular reflex tests, which represent a standardized method for diagnosing CAN. By assessing this correlation, we aim to gain a deeper understanding of how sudomotor function, reflecting the activity of the autonomic nervous system, correlates with the results of cardiovascular reflex tests, which measure the responses of the autonomic nervous system. This evaluation could provide valuable insights into the functional status of the patient's autonomic nervous system and help identify CAN early. By assessing this correlation, we can also validate the use of sudomotor function as a non-invasive and accessible method for detecting CAN, which could be particularly useful in routine clinical practice and large-scale epidemiological studies. Ultimately, the results of this study have significant implications for the diagnosis of CAN, contributing to the improvement of patient care for this condition.

## Materials and methods

This cross-sectional study was conducted between June 2019 and June 2020. The Ethics Committee of Nicolae Malaxa Clinical Hospital, Bucharest, Romania, granted ethical approval for the study (approval number: 2145). All data were obtained according to the hospital's standard of care for patients with T2DM. All patients included in the study provided informed consent for data collection and secondary use of medical data for research purposes.

Study population 

The inclusion criteria encompassed patients who had consultations at the hospital outpatient department during the study period, patients who signed informed consent, those diagnosed with T2DM, and overweight/obese individuals aged above 18 years. Conversely, exclusion criteria included patients who did not sign informed consent, those with other diabetes types (type 1 diabetes, latent autoimmune diabetes of adults, maturity-onset diabetes of the young), individuals aged 18 years and younger, pregnant women, patients diagnosed with neoplasms within the past five years, those with stroke sequelae, history of myocardial infarction, pelvic limb amputations, pre-existing chronic kidney disease predating diabetes diagnosis, and patients with neuropathy caused by alternative factors (alcoholism, vitamin B12 deficiency). We excluded patients with stroke sequelae from the study because we anticipated potential false positive results in sensitivity tests or Sudoscan (Impeto Medical, Moulineaux, France). Additionally, we excluded patients with a recent history of neoplasms due to the risk of neuropathy development in the context of chemotherapy. Patients with limb amputations were also excluded because the Sudoscan test cannot be conducted in such cases.

Examination of patients

Data on anthropometric indices, including height, weight, body mass index (BMI), waist circumference, waist-to-hip ratio, blood pressure values in supine and orthostatic positions, heart rate in supine and orthostatic positions, and smoking status, were recorded.

Measurement of biochemical parameters

The following samples were collected from venous plasma after eight hours of fasting: serum glucose, glycated hemoglobin HbA1c, total cholesterol, high-density lipoproteins cholesterol (HDLc), low-density lipoprotein (LDL) cholesterol, triglycerides, bilirubin, C-reactive protein, serum creatinine, potassium, magnesium, chloride, sodium, calcium, urea, glutamic oxaloacetic transaminase (GOT), glutamic pyruvic transaminase (GPT), gamma-glutamyl transferase (GGT), and urinary albumin-to-creatinine ratio (ACR).

Diagnosis of CAN

The evaluation of CAN involved conducting an ECG and assessing the QTc interval in the morning prior to medication intake. CARTs were also performed, encompassing assessments of heart rate variability during deep breathing, the Valsalva maneuver, and orthostatic changes. Furthermore, orthostatic systolic and diastolic blood pressure measurements will be conducted during an isometric effort. The results of CARTs were categorized as usual if no abnormal findings were detected. They were considered to indicate mild dysfunction if one of the five tests was abnormal, moderate dysfunction if two or three were abnormal, and severe dysfunction if more than three were abnormal. All these measurements were performed using the ESP-01-PA Ewing Tester neuropathic measuring and analyzing system with an ECG module. At the same time, the diagnosis of CAN was evaluated by performing a sweat test using Sudoscan assessment. The patient places their hands and feet on the electrodes during the test. The test takes three minutes to perform, is painless, and requires no subject preparation. Additionally, Sudoscan incorporates built-in algorithms that integrate electrochemical skin conductance with age to generate a score estimating the current risks of CAN (Sudoscan-CAN score).

The statistical analysis of the population was conducted using IBM SPSS Statistics for Windows, Version 20.0 (Released 2011; IBM Corp., Armonk, New York, United States). Continuous variables usually distributed were presented as mean ± SD (standard deviation), and non-normal variables were expressed as median (interquartile range (IQR)). In contrast, categorical variables were reported as absolute counts and percentages. Statistical significance was determined at a 95% confidence interval. Analysis of variance (ANOVA) was employed to compare groups for quantitative variables. At the same time, the χ2 test was utilized for categorical variables-multiple linear regression was used to estimate the independent correlation of the Sudoscan-CAN score with Ewing Tests.

## Results

The study included 271 subjects with 52% (n=143) being female. Significant statistical differences were observed between female and male subjects regarding age, systolic blood pressure, fasting blood glucose, HbA1c level, total cholesterol, triglycerides, gamma-glutamyl transferase, and bilirubin levels (Table 1, 2). It appears that women have better glycemic control than men, but men have better control over total cholesterol. Additionally, GGT and bilirubin values were significantly higher among men.

**Table 1:** Anthropometric and biochemical parameters in the studied cohort stratified by gender F= female, M= male, SD= standard deviation, BMI= body mass index, BP= blood pressure, HR= heart rate, FPG= fasting plasmatic glucose, HbA1c = glycated hemoglobin, TC = total cholesterol, HDL=high-density lipoproteins, LDL- low-density lipoproteins, eGFR= estimated glomerular filtration rate, CRP= c-reactive protein Statistical significance, p<0.05

Parameters	Female (n=143)		Male (n=128)		Total (n=271)		
	Mean	SD	Mean	SD	Mean	SD	P-value
Age (years)	63.31	8.58	59.67	9.25	61.59	9.07	<0.001
BMI (kg/m²)	32.66	5.28	31.74	5.35	32.23	5.32	0.16
Systolic BP (mmHg)	132.56	19.63	136.33	16.38	134.34	18.24	<0.001
Dyastolic BP (mmHg)	74.59	10.85	78.59	10.37	76.48	10.79	0.41
HR (bpm)	74.56	10.99	75.62	10.18	75.06	10.61	0.41
FPG (mg/dl)	180.70	81.94	198.29	85.25	189.01	83.83	0.03
HbA1c (%)	7.86	1.62	8.35	2.05	8.09	1.85	0.01
TC (mg/dl)	206.35	58.62	188.76	49.36	198.01	55.03	0.01
HDL-c (mg/dl)	52.41	12.84	48.18	13.63	50.42	13.36	0.80
TC/HDL-c	4.13	1.55	4.18	1.49	4.15	1.52	0.02
LDL-c (mg/dl)	115.95	50.41	101.49	46.59	109.11	49.09	0.75
Urea (mg/dl)	40.08	20.24	40.74	17.53	40.39	19.00	0.01
Creatinine (mg/dl)	0.87	0.27	1.08	0.33	0.97	0.32	0.59
eGFR (ml/min/1,73m²)	77.18	27.12	77.13	22.26	77.16	24.89	0.21
Bilirubin (mg/dl)	0.59	0.25	0.73	0.39	0.66	0.33	0.04
B12 vitamin (pg/ml)	386.26	187.94	476.56	254.40	430.19	224.31	0.23
CRP (mg/dl)	1.38	2.54	0.60	0.90	0.99	1.95	0.99

**Table 2:** Biochemical parameters in the studied cohort stratified by gender TGL = triglycerides, ACR= Albumin-to-creatinine ratio, GOT = glutamic oxaloacetic transaminase, GPT = glutamic pyruvic transaminase, GGT= gamma-glutamyl transferase Statistical significance, p<0.05

	Female (n=143)		Male (n=128)		Total (n=271)		
Parameters	Median	95% CI	Median	95% CI	Median	95% CI	P-value
Diabetes duration (years)	7	6 - 9	6.5	5-8	7	6 - 9	0.49
TGL (mg/dl)	161.5	140.5 - 191.2	186	160 - 231	173	154 - 192	0.03
ACR (mg/g)	23.75	17.42 - 34.68	21.3	9.66 - 35.98	23	17.11 – 33.24	0.12
GOT (UI/L)	20.5	19.5 - 25	24	21 - 32	22	20 - 25	0.01
GPT (UI/L)	25	23 - 27	30	24 - 37	27	24 - 28	0.12
GGT [UI/L]	29	23 - 38	44	31 - 64	34	28 - 42	0.03

From the entire patient cohort, 81.1% (n=220) were identified with CAN (CAN +), and 51 patients without CAN served as the control group (CAN-). Among the CAN+ group, there were 40.92% mild cases (n=90), 47.27% were moderate cases (n=104), and 11.81% were severe cases (n=26). Within the CAN+ group, 54% (n=119) were women (Table 3).

**Table 3:** Gender distribution of groups CAN = cardiac autonomic neuropathy

Gender	CAN (–)		CAN (+)		Total
		Mild	Moderate	Severe	
Female	24	46	59	14	143
Male	27	44	45	12	128
Total	51	90	104	26	271

The mean age for the mild CAN group was 63.56 ± 8.45 years, for the moderate CAN group it was 61.00 ± 9 years, for the severe CAN group it was 59.50 ± 7.94 years, and for the control group it was 60.20 ± 9.83 years. Regarding the HbA1c and FPG levels, they increased directly proportional to the severity of CAN, with a mean value of 8.86 ± 2.21 among patients with severe CAN. The total cholesterol and triglyceride levels registered significantly lower values among mild CAN patients. Patients with severe CAN exhibited higher albumin-to-creatinine ratio (ACR) values compared to those with mild or moderate CAN (Table 4).

**Table 4:** Anthropometric measurements of the groups and laboratory parameters WC = waist circumference; Pv-1 = p-value comparing mild CAN with CAN-, Pv-2 = p-value comparing moderate CAN with CAN-, Pv-3 = p-value comparing severe CAN with CAN-

Parameters	CAN (–) (N = 51), mean±SD	CAN (+), mean±SD			p-values		
		Mild (n =90 )	Moderate (n =104 )	Severe (n =26 )	Pv-1	Pv-2	Pv-3
Age (year)	60.20 ± 9.83	63.56 ± 8.45	61.00 ± 9	59.50 ± 7.94	.004	.592	.216
BMI (kg/m²)	30.27 ± 4.18	33.24 ± 5.74	32.95 ± 5.43	33.12 ± 5.24	.012	.265	.371
Height (cm)	167.88 ± 9.2	164.81 ± 10.07	164.48 ± 7.4	164.96 ± 9.06	.193	.269	.656
WC (cm)	101.90 ± 10.2	107.11 ± 13.61	108.26 ± 13.18	108.08 ± 12.48	.167	.107	.331
FPG (mg/dL)	178.84 ± 70.2	185.77 ± 93.73	191.87 ± 64	229.81 ± 108.46	.614	.780	.009
HbA1c (%)	7.67 ± 1.57	8.13 ± 2.06	8.31 ± 1.49	8.86 ± 2.21	.793	.324	.026
TC (mg/dL)	203.40 ± 49.9	189.40 ± 51.51	203.69 ± 66.69	202.85 ± 56.89	.042	.398	.638
HDL-C (mg/dL)	49.78 ± 13.4	49.93 ± 12.66	52.44 ± 14.8	50.26 ± 13.19	.636	.214	.951
TC/HDL-C	4.38 ± 1.62	3.96 ± 1.29	4.14 ± 1.74	4.20 ± 1.51	.100	.920	.877
LDL-C (mg/dL)	116.80 ± 48.6	104.40 ± 44.69	108.18 ± 57.07	105.32 ± 50.28	.212	.881	.679
TGL (mg/dL)	213.31 ± 136.39	190.85 ± 44.69	237.48 ± 195.94	254.97 ± 126.68	.043	.176	.126
Creatinine (mg/dl)	0.93 ± 0.25	0.99 ± 121.17	0.95 ± 0.32	1.04 ± 0.34	.416	.672	.227
ACR (mg/g)	28.23 ± 91.4	58.01 ± 0.36	72.31 ± 122.78	168.93 ± 435.19	.771	.707	.005
AST (U/L)	24.77 ± 12.3	26.64 ± 14.1	30.34 ± 18.21	26.09 ± 12.69	.936	.040	.815
ALT (U/L)	29.25 ± 15.9	30.03 ± 19.78	35.39 ± 20.5	32.21 ± 15.33	.466	.053	.744
GGT (U/L)	64.20 ± 103.6	43.85 ± 39.17	85.80 ± 106.78	55.68 ± 61.79	.094	.070	.815

Electrochemical skin conductance was lower in the CAN+ group than the CAN- group at hands (67.34±15.51μS versus 72.38±12.12μS, p=0.008) and feet (73.37±13.38μS versus 82.84 ±10.29μS, p<0.001). The mean SUDOSCAN-CAN score was 36.88±8.69 in the CAN+ group and 26.02±36.88 in the CAN- groups, respectively (p<0.001).

Correlation of Sudoscan-CAN score with Ewing scores 

**Figure 1:**
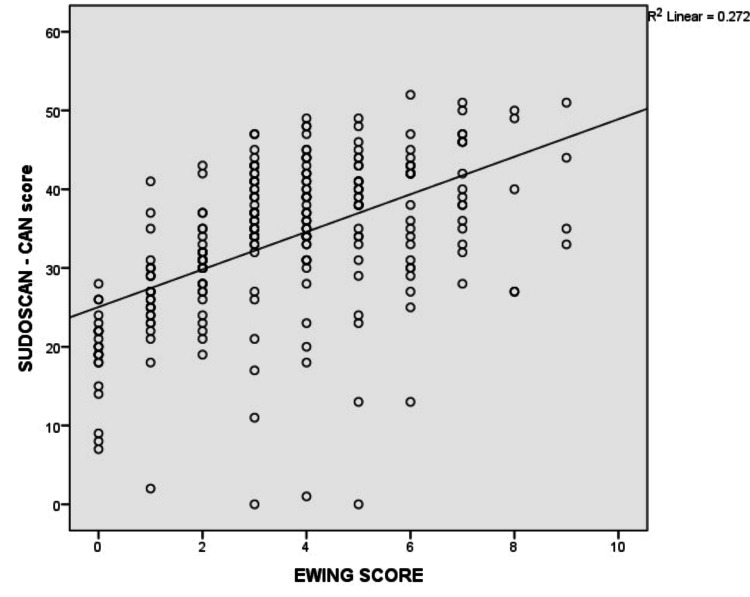
Scatterplot showing the relationship between Sudoscan-CAN score on y-axis and Ewing score on x-axis CAN = cardiac autonomic neuropathy

The scatterplot shows the relationship between the Sudoscan-CAN score and the Ewing score. Sudoscan-CAN score showed a significantly positive correlation with Ewing scores (r= 0.522, p<0.001) (Figure 1).

On multiple linear regression, the Sudoscan-CAN score remained significantly associated with age, high BMI, long-standing diabetes, and Ewing score (Table 5).

**Table 5:** Clinical factors associated with Sudoscan score in patients with type 2 diabetes using multiple linear regression BMI = body mass index

	Standard β-coefficient	P-value
Age	.256	<0.001
BMI	.430	<0.001
Diabetes duration	.074	.01
Ewing score	.412	<0.001

Performance of Sudoscan in detecting CAN

**Figure 2:**
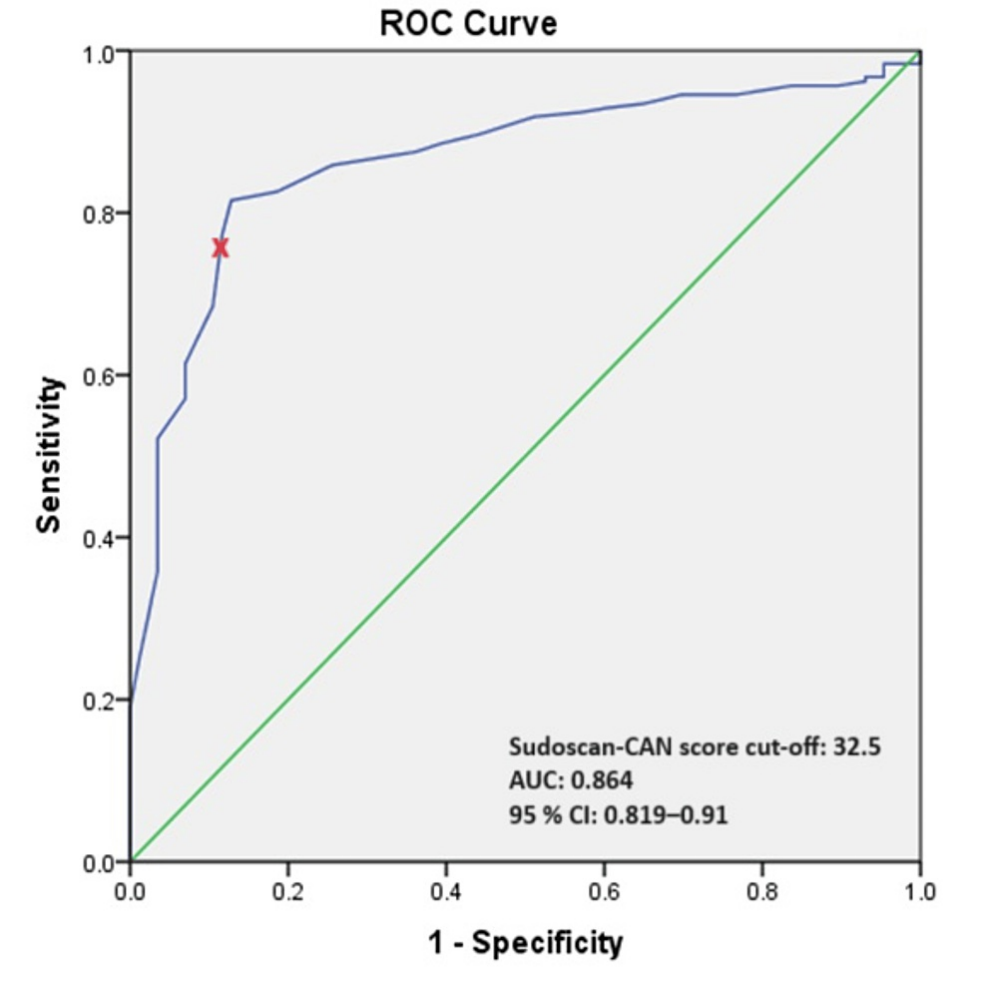
ROC curve of Sudoscan-CAN score in detecting CAN patients with T2DM Diagonal segments are produced by ties. CAN = cardiac autonomic neuropathy, T2DM = type 2 diabetes mellitus; ROC = receiver operating characteristic, AUC = area under the curve

The area under the receiver operating characteristic (ROC) curve of the Sudoscan-CAN score to predict CAN was 0.864 (OR 0.87 (95%CI 0.819-0.91) of the total square (Figure 2). The Sudoscan-CAN score cut-off was 32.5, and the test had 81.5% sensitivity and 12.8% specificity to detect CAN with a positive predictive value (PPV) of 84% and a negative predictive value (NPV) of 69%. 

## Discussion

CAN represents an autonomous risk factor for cardiovascular and cerebrovascular diseases, exacerbating mortality rates among individuals with diabetes. The American Diabetes Association (ADA) recommends screening for neuropathy symptoms beginning five years after type 1 diabetes mellitus diagnosis and at the time of T2DM diagnosis because patients with T2DM can experience a silent progression, and at the time of diagnosis, a significant proportion of them already have associated chronic complications [16]. However, the complexity of diagnostic assessments, diverse criteria, and a paucity of pertinent studies contribute to CAN being among the most overlooked complications in diabetes management.

Given the increasing incidence of diabetes, CAN is a common complication, starting early in T2DM and having multiple and novel correlations. These correlations may include intestinal microbiome, cytokine levels and inflammation, oxidative stress, obstructive sleep apnea, and endothelial dysfunction. Patients at higher risk of CAN are those with older age, longer duration of disease, poor glycemic control, as well as those with microvascular complications (diabetic polyneuropathy, retinopathy, and nephropathy), and other cardiovascular risk factors, especially obesity, dyslipidemia, and hypertension in T2DM. In the current study, patients with elevated Sudoscan-CAN scores have a 17-fold higher probability of having CAN than those with lower scores. Factors such as age, duration of diabetes, glycemic control, and other comorbidities can influence both Sudoscan-CAN scores and the presence of CAN. Controlling for these variables in statistical analyses strengthens the validity of the observed association. Based on our findings, we conclude that Sudoscan can be helpful in detecting patients at risk of having CAN, and considering that this method is non-invasive and the portable nature of Sudoscan, it can be used in educational programs as part of a CAN screening program. 

Sudomotor function, a subset of autonomic function, indicates the integrity of sympathetic nerve fibers that innervate the sweat glands. In individuals with diabetes, sudomotor dysfunction is characterized by a diminished sweat response to elevated ambient temperature and humidity, particularly noticeable in the lower extremities [17]. Prolonged diabetes leads to chronic impairment of sudomotor function, resulting in decreased skin flexibility, dryness, and fissures, especially in the feet. The C fibers innervate the sweat glands and are typically thin, long, and poorly myelinated, rendering them highly vulnerable to damage from metabolic processes. Studies have shown that the density of nerve fibers innervating sweat glands, as determined by skin biopsies, correlates with glycemic measures such as HbA1c levels in diabetic patients [18]. Furthermore, nerve damage may occur prior to the onset of clinically evident diabetes, as evidenced by reports of sudomotor dysfunction in individuals with impaired glucose tolerance and metabolic syndrome [19,20]. Sudomotor dysfunction, a hallmark of autonomic neuropathy in diabetes, can manifest clinically in various ways. Patients may experience reduced or absent sweating, particularly in the lower extremities, leading to dry skin, decreased skin elasticity, and an increased risk of developing foot complications such as ulcers and infections. Moreover, sudomotor dysfunction can impair temperature regulation, making individuals more susceptible to heat intolerance or fluctuations in body temperature. By elucidating the clinical manifestations and prognostic implications of sudomotor dysfunction, clinicians can better recognize and manage this aspect of diabetic neuropathy, thereby potentially mitigating the risk of associated complications and improving patient care.

Following the Sudoscan test, a cardiovascular risk score is obtained [21]. In the current study, this score was statistically significantly associated with the presence of CAN. Multiple regression analysis, which included significant variables, showed an independent association of the risk score for CAN obtained from the sweat test with age, duration of diabetes, BMI, and the Ewing score.

Future research should focus on longitudinal and validation studies across diverse populations to confirm the utility of Sudoscan in CAN screening and management. Identifying novel biomarkers and refining screening protocols could enhance early detection and intervention strategies, ultimately improving outcomes for individuals with diabetes.

Limit

We acknowledge several limitations in our study. Firstly, although our cross-sectional cohort had a relatively small sample size, further prospective evaluation is required to validate the clinical utility of Sudoscan in assessing risk and predicting outcomes in CAN. Secondly, the enrollment of patients from a single center prevents us from definitively establishing a cause-and-effect relationship between Sudoscan CAN scores and other diabetes complications. The single-center design of our study may introduce selection bias, potentially resulting in differences between included and excluded patients that could impact the findings. Additionally, the limited sample representation hinders the generalizability of our results to populations with diverse characteristics or different medical settings. To mitigate these limitations in future research, multicenter evaluations are recommended, which would allow for a more comprehensive representation of the population and enable the assessment of result variability across various medical settings. Furthermore, implementing stricter control and reporting of confounding factors could improve the validity and interpretation of the findings.

## Conclusions

CAN represents a manageable complication of T2DM, posing an elevated risk of cardiovascular mortality. Despite its significance, CAN diagnosis is often challenging, leading to underrecognition. Sudoscan demonstrates promise in identifying individuals at heightened risk of CAN, offering a non-invasive, portable, and user-friendly screening tool suitable for outreach programs or resource-limited settings. By facilitating early detection, Sudoscan may enable timely interventions to mitigate cardiovascular risk, thereby potentially impacting clinical practice. Future research should focus on overcoming current limitations to further validate Sudoscan's utility, including assessing its predictive value through longitudinal studies and evaluating cost-effectiveness. Such endeavors would enhance our understanding of Sudoscan's effectiveness and its potential to improve outcomes in individuals with T2DM.

## References

[1] Zilliox LA, Russell JW (2020). Is there cardiac autonomic neuropathy in prediabetes?. Auton Neurosci.

[2] Achmad C, Lim NS, Pramudyo M, Iqbal M, Karwiky G, Febrianora M, Natalia N (2023). Relation between glycemic control and cardiac autonomic neuropathy in patients with diabetes mellitus type 2. Curr Probl Cardiol.

[3] Yuan T, Li J, Fu Y (2018). A cardiac risk score based on sudomotor function to evaluate cardiovascular autonomic neuropathy in asymptomatic Chinese patients with diabetes mellitus. PLoS One.

[4] Kaur D, Tiwana H, Stino A, Sandroni P (2021). Autonomic neuropathies. Muscle Nerve.

[5] Sudo SZ, Montagnoli TL, Rocha BS, Santos AD, de Sá MP, Zapata-Sudo G (2022). Diabetes-induced cardiac autonomic neuropathy: impact on heart function and prognosis. Biomedicines.

[6] Spallone V, Ziegler D, Freeman R (2011). Cardiovascular autonomic neuropathy in diabetes: clinical impact, assessment, diagnosis, and management. Diabetes Metab Res Rev.

[7] Vinik AI, Ziegler D (2007). Diabetic cardiovascular autonomic neuropathy. Circulation.

[8] Stephenson J, Fuller JH (1994). Microvascular and acute complications in IDDM patients: the EURODIAB IDDM complications study. Diabetologia.

[9] Buse JB, Bigger JT, Byington RP (2007). Action to control cardiovascular risk in diabetes (ACCORD) trial: design and methods. Am J Cardiol.

[10] Abboud FM, Singh MV (2017). Autonomic regulation of the immune system in cardiovascular diseases. Adv Physiol Educ.

[11] Hillis GS, Woodward M, Rodgers A (2012). Resting heart rate and the risk of death and cardiovascular complications in patients with type 2 diabetes mellitus. Diabetologia.

[12] Magkas N, Tsioufis C, Thomopoulos C (2019). Orthostatic hypotension: From pathophysiology to clinical applications and therapeutic considerations. J Clin Hypertens (Greenwich).

[13] Spallone V (2018). Blood pressure variability and autonomic dysfunction. Curr Diab Rep.

[14] Călin P, Viorel M, Luchiana P, Mihaela C, Lavinia P (2022). Masked nocturnal hypertension as a result of high prevalence of non-dippers among apparently well-controlled hypertensive patients with type 2 diabetes mellitus: data from a prospective study. Diabetol Metab Syndr.

[15] He T, Wang C, Zuo A (2017). Electrochemical skin conductance may be used to screen for diabetic cardiac autonomic neuropathy in a Chinese population with diabetes. J Diabetes Res.

[16] (2024). 12. Retinopathy, neuropathy, and foot care: standards of care in diabetes-2024. Diabetes Care.

[17] Fealey RD, Low PA, Thomas JE (1989). Thermoregulatory sweating abnormalities in diabetes mellitus. Mayo Clinic Proc.

[18] Luo KR, Chao CC, Hsieh PC, Lue JH, Hsieh ST (2012). Effect of glycemic control on sudomotor denervation in type 2 diabetes. Diabetes Care.

[19] Sun K, Liu Y, Dai M (2012). Accessing autonomic function can early screen metabolic syndrome. PLoS One.

[20] Yang Z, Xu B, Lu J (2013). Autonomic test by EZSCAN in the screening for prediabetes and diabetes. PLoS One.

[21] Gavan DE, Gavan A, Bondor CI, Florea B, Bowling FL, Inceu GV, Colobatiu L (2022). SUDOSCAN, an innovative, simple and non-invasive medical device for assessing sudomotor function. Sensors (Basel).

